# Isolation of intact sub-dermal secretory cavities from *Eucalyptus*

**DOI:** 10.1186/1746-4811-6-20

**Published:** 2010-09-01

**Authors:** Jason QD Goodger, Allison M Heskes, Madeline C Mitchell, Drew J King, Elizabeth H Neilson, Ian E Woodrow

**Affiliations:** 1 School of Botany, The University of Melbourne, Parkville, Victoria 3010, Australia

## Abstract

**Background:**

The biosynthesis of plant natural products in sub-dermal secretory cavities is poorly understood at the molecular level, largely due to the difficulty of physically isolating these structures for study. Our aim was to develop a protocol for isolating live and intact sub-dermal secretory cavities, and to do this, we used leaves from three species of *Eucalyptus *with cavities that are relatively large and rich in essential oils.

**Results:**

Leaves were digested using a variety of commercially available enzymes. A pectinase from *Aspergillus niger *was found to allow isolation of intact cavities after a relatively short incubation (12 h), with no visible artifacts from digestion and no loss of cellular integrity or cavity contents. Several measurements indicated the potential of the isolated cavities for further functional studies. First, the cavities were found to consume oxygen at a rate that is comparable to that estimated from leaf respiratory rates. Second, mRNA was extracted from cavities, and it was used to amplify a cDNA fragment with high similarity to that of a monoterpene synthase. Third, the contents of the cavity lumen were extracted, showing an unexpectedly low abundance of volatile essential oils and a sizeable amount of non-volatile material, which is contrary to the widely accepted role of secretory cavities as predominantly essential oil repositories.

**Conclusions:**

The protocol described herein is likely to be adaptable to a range of *Eucalyptus *species with sub-dermal secretory cavities, and should find wide application in studies of the developmental and functional biology of these structures, and the biosynthesis of the plant natural products they contain.

## Background

The application to plants of systems biology technologies, such as metabolomics and transcriptomics, has generally followed a non-targeted approach using bulk material composed of numerous tissue types [1]. Attempts to focus on particular plant tissues and cells have raised a number of significant technical and experimental challenges, including the isolation of sufficient quantities of a pure tissue or cell type. Recent experimental developments have enabled fine scale analyses of tissues and even single cells in some cases [2,3]. Nevertheless, many biochemically important plant tissues and cell types remain extremely difficult to isolate in sufficient quantities.

The specialised secretory structures in which essential oils (mono- and sequiterpenes) are synthesised and stored are ideal candidates for fine scale application of systems biology techniques [4]. The oils are secreted and stored in a variety of structures ranging from single-cell idioblasts, such as those found in lemongrass [5], to multicellular glandular secretory complexes such as the superficial trichomes of mint and sage [6,7], the sub-dermal ducts of conifers and the sub-dermal cavities characteristic of *Eucalyptus *and *Citrus *species [8,9]. Such essential oil-bearing structures not only contain specialised biosynthetic cells that can produce a range of important natural products, but they are also discretely separated within plants, numerous in many species and importantly possess unique biochemical features [10].

Recent advances in elucidating the pathways for essential oil biosynthesis have come with the application of transcriptomics to isolated glandular trichomes from species such as peppermint [11], sweet basil [12], tobacco [13,14], sweet wormwood [15] and hop [16]. In general, this work has used an expressed sequence tag approach to try to identify the relevant biosynthetic enzymes using isolated glandular trichomes as a source of mRNA from which trichome-derived cDNA libraries have been produced. Such research has been aided greatly by the fact that trichomes protrude from the plant surface and can therefore be isolated in relatively large quantities, facilitating analysis of the trichome metabolome, proteome and transcriptome [17]. Techniques used to harvest trichomes are mostly modifications of the method of glass bead abrasion originally developed for mint species by Gershenzon and co-authors [18], but non-abrasive techniques for physically removing trichomes have also been developed [13,14,16].

The internally embedded secretory structures have proven much more difficult to isolate from the surrounding tissues, and consequently have not been as well studied at the molecular level. The most advanced work has been on the monoterpenes and diterpene resin acids (collectively oleoresin) housed in the resin ducts of conifers [19,20]. This work has been aided by the availability of extensive conifer genomics resources, and the recent development of a method to separate duct complexes from surrounding tissue using laser microdissection [20]. In contrast, no method has been developed to isolate sub-dermal secretory cavities from the essential oil producing and therefore economically important species of the Rosaceae (*Rosa *spp.), Rutaceae (*Citrus *spp.) or Myrtaceae (*Eucalyptus *and *Melaleuca *spp.). The multicellular secretory structures in these families are generally ellipsoidal in shape and consist of a sub-epidermal lumen (the repository for essential oils) delimited by an internal layer of flattened, thin-walled secretory cells, and an external layer of thicker-walled parenchymatous cells [21].

The essential oils extracted from the secretory cavities of a number of *Eucalyptus *species have long been of economic value as pharmaceuticals and as fragrance additives [22], but they have recently received increased attention for their anti-microbial [23] and other medicinal properties [24,25]. Many questions regarding secretory cavity development remain unanswered (see review by [6]), and given the ontogenetic, structural and compositional distinction between superficial glandular trichomes, resin ducts and sub-dermal cavities, lessons learnt from one type of secretory structure may not necessarily be applicable to the others.

We aimed to develop a protocol to isolate live *Eucalyptus *sub-dermal secretory cavities free from all surrounding leaf mesophyll tissues, without compromising the integrity of the cells bounding the lumen and without the loss of any lumen contents. The relatively large sub-dermal secretory cavities of *Eucalyptus *species not only house economically important essential oils, but these structures have recently been shown to contain other, non-volatile natural products [26], making them excellent candidates for biosynthetic studies. In particular, we selected three species with highly abundant secretory cavities: *Eucalyptus globulus *Labill. ssp. *globulus *(Tasmanian blue gum), the world's major source of the pharmaceutical monoterpene 1,8-cineole [27], *E. polybractea *R.T. Baker (blue mallee), Australia's key commercial source of 1,8-cineole [22], and a non-commercially harvested species, *E. froggattii *Blakely (kamarooka mallee) the secretory cavities of which contain substantially lower levels of monoterpenes relative to sesquiterpenes [26].

## Results

### Secretory cavity isolation following enzymatic digestion

Secretory cavities were isolated from surrounding leaf tissues following partial enzymatic digestion of leaves. Six commercially available enzymes were trialed at amounts ranging from 50 to 250 units ml^-1 ^buffer. Two enzymes were from *Aspergillus niger*: pectinase PASE (Worthington, Lakewood, NJ) and pectinase in glycerol P-4716 (Sigma, St. Louis, MO); two were from *Rhizopus*: pectinase P-2401 (Sigma) and Calbiochem macerase-pectinase (Merck, Darmstadt, Germany); and two were from *Trichoderma viride*: cellulase C-1794 (Sigma) and cellulase 'Onozuka' RS (Yakult Pharmaceutical, Tokyo, Japan). Fresh leaves were cut into 2 mm strips and placed in 'Standard' buffer (500 mM sorbitol, 5 mM MES-KOH, 1 mM CaCl_2_, pH 5.5) containing each enzyme and incubated for between 12 and 24 h at temperatures as per manufacturer's instructions. To maximize the chance of isolating cavities with live cells, 24 h was chosen as the maximum allowable incubation time.

**Figure 1:**
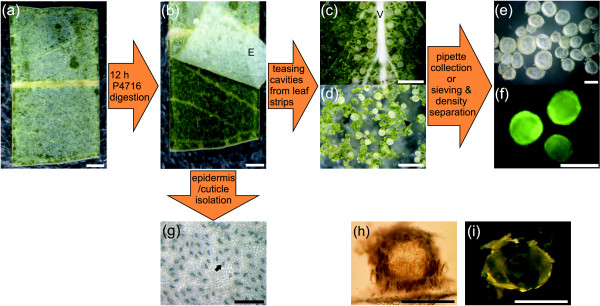
**Flow chart of the successful secretory cavity isolation protocol**. (a) Leaf strip dissected from a *Eucalyptus polybractea *leaf. (b) Leaf strip after 12 h digestion with *Aspergillus *P-4716 pectinase (Sigma) and peeling away of the epidermis (E) with attached cuticle. (c) Digested leaf strip with removed epidermis showing freed cavities (arrow) and vasculature (V). (d) Mixture of isolated cavities (arrow) and mesophyll tissue (arrowhead) after leaf strip was teased apart with forceps. (e) Secretory cavities freed from the mesophyll tissue in which they were embedded. Isolated cavities can be collected via pipette or for larger numbers of cavities, after sieving and density separation steps. (f) Autofluorescence of isolated cavities imaged under UV illumination with GFP2 filter set show no evidence of characteristic red chlorophyll fluorescence. (g) Isolation of the epidermis is an added advantage of the protocol that may be of use in examinations of cuticular or epidermal properties such as stomatal (arrow) arrangement. (h) An incompletely isolated cavity using *Rhizopus *pectinase (Sigma). (i) An over-digested cavity using *Trichoderma *cellulose (Yakult). Scale bars represent 1 mm for panels a to d, and 200 μm for panels e to i.

Digestion was deemed complete when the leaf epidermis with attached cuticle could be readily peeled away and secretory cavities teased apart from the remaining leaf tissues using fine forceps under a dissecting microscope. The digestion of leaf strips with either of two pectinases from *Aspergillus *at 250 units ml^-1 ^standard buffer produced numerous isolated secretory cavities (free from mesophyll cells) from each leaf strip within minutes of teasing leaf tissues apart (Table 1; Fig. 1). Although both *Aspergillus *pectinases were successful, Sigma P-4716 yielded predominantly mesophyll-free cavities in only 12 h whereas Worthington PASE required 16-20 h to isolate a lower proportion of totally mesophyll-free cavities. Any digested cavities that still had small amounts of mesophyll or vasculature tissue attached could generally be cleaned of such cells by gentle brushing with a 1 μm tip microprobe (World Precision Instruments Pty Ltd, Sarasota, FL). In addition to lower cavity yields, incubation with Worthington PASE resulted in browning of the mesophyll cells in the leaf strips during incubation, which subsequent analysis following the method of Vernon (1960) [28] showed was due to the conversion of chlorophylls to phaeophytins. Although not necessarily important when isolating secretory cavities, this latter observation was deemed indicative of potential artifacts of the digestion process when using Worthington PASE. Both *Rhizopus *pectinases produced semi-isolated cavities with mesophyll cells still attached (Fig. 1h) and these cells could not be readily removed with gentle microprobe brushing. Therefore the *Rhizopus *enzymes were incapable of successfully isolating mesophyll cell-free cavities, at least with incubations of 24 h or less. The two *Trichoderma *cellulases were similarly unsuccessful, but for a different reason. Digestion with these enzymes for 24 h resulted in almost complete digestion of all leaf tissues, including the cells bounding secretory cavity lumena, and therefore the loss of cavity structural integrity (Fig. 1i). Incubation with the *Rhizopus *or *Trichoderma *enzymes for less than 24 h was insufficient to enable the epidermis and cuticle to be easily removed, thereby making cavities inaccessible.

**Table 1:** Isolation of embedded *Eucalyptus *secretory cavities via enzymatic digestion.

Enzyme (Manufacturer)	Source	Time (h)^‡^	Quality of isolation	Comments
Pectinase P-4716 (Sigma)	*Aspergillus niger*	12	Excellent	Successful isolation of cavities free from mesophyll cells. Mesophyll cells remain green.
Pectinase PASE (Worthington)	*Aspergillus niger*	16	Good	Mostly successful isolation of cavities free from mesophyll cells. Mesophyll cells become brown.
				
Pectinase P-2401 (Sigma)	*Rhizopus *spp.	24	Poor	Mesophyll cells generally remain attached to cavities. Mesophyll cells become brown.
Macerase-pectinase 'Calbiochem' (Merck)	*Rhizopus *spp.	24	Poor	Mesophyll cells generally remain attached to cavities. Mesophyll cells become brown.
				
Cellulase C-1794 (Sigma)	*Trichoderma viride*	24	Poor	Cavities lose structural integrity. Lumen contents become brown. Mesophyll cells become brown.
Cellulase 'Onozuka RS' (Yakult)	*Trichoderma viride*	24	Poor	Cavities lose structural integrity. Lumen contents become yellow. Mesophyll cells become brown.

^‡ ^Minimum digestion time required to remove epidermis with attached cuticle and isolate secretory cavities.

After successful enzymatic digestion with Sigma P-4714 (250 units ml^-1 ^standard buffer; 25°C), a pipette was used to collect each individual cavity from within the milieu of mesophyll cells and vasculature (see Fig. 1). To simplify the collection of hundreds to thousands of cavities, the protocol was extended to include sieving and density separation steps. Once the epidermis and cuticle had been removed from digested leaf strips and leaf tissues teased apart (see Fig. 1), isolated cavities were separated by sieving the mixture of tissues and cells with a coarse sieve to remove large pieces of epidermis/cuticle and vasculature and then retaining the isolated cavities and similar-sized clumps of leaf tissue on a 140 μm diameter stainless steel mesh, whilst removing individual cells and small debris. Isolated cavities were then separated from the other retained material by density centrifugation. Cavities accumulated at the interface between standard buffer and 1 M sucrose in standard buffer following centrifugation (300 *g *for 5 min). Intact cavities were collected, washed with standard buffer and concentrated (100 *g *for 10 min).

### Structural and metabolic properties of cavities isolated using Sigma P-4716

**Figure 2:**
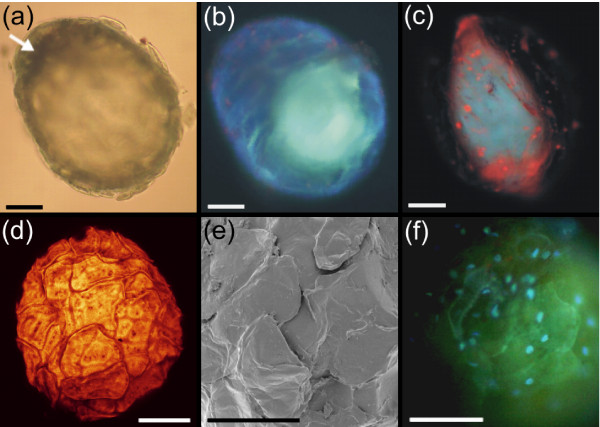
**Application of microscopy techniques to essential oil secretory cavities isolated from *Eucalyptus polybractea *leaves**. (a) Isolated cavity viewed under transmitted light, the arrow indicates the estimated extent of the lumen and the approximate position of microprobe insertion in panel c. (b) Isolated cavity viewed with UV excitation (UV filter) showing green autofluorescence of the non-volatile resinous material. (c) Punctured cavity stained with nile red and viewed under UV excitation (UV filter), remaining essential oils fluoresce red, but the resinous component (green autofluorescence) remains unstained. (d) Confocal micrograph of an isolated cavity showing outer cell arrangement. (e) Scanning electron micrograph detailing outer cavity cells. (f) Isolated cavity viewed under UV excitation (UV filter) after Hoechst vital staining showing the integrity of cavity cell nuclei (bright blue fluorescence) and the green autofluorescence of the resinous component within the lumen. All scale bars represent 50 μm.

Photosynthetic pigments were extracted and quantified as indicators of mesophyll cell contamination in triplicate collections of 50 isolated cavities. The results were compared to those for sections of digested leaf dissected to encompass 50 cavities. Total chlorophyll (mean ± s.e.) was 173.42 ± 20.28 ng cavity^-1 ^in dissected leaf sections post digestion, but only 2.98 ± 0.46 ng cavity^-1 ^for isolated cavities, suggesting very few mesophyll cells remained attached to isolated cavities. Moreover, the distinct lack of chlorophyll autofluorescence observed under UV microscopy (see Figs. 1f, 2b, c &2f), suggests that the cells bounding the cavity lumen possess few, if any, chloroplasts. The isolated cavities were shown to be amenable to advanced microscopy techniques such as confocal microscopy (Fig. 2d) and scanning electron microscopy (SEM; Fig. 2e). The application of these techniques clearly provides information on cavity cell arrangement, with the outer cavity cells flattened, rather than parenchymatous, and arranged in an overlapping manner. The isolation protocol did not disrupt the structural integrity of the secretory cavities and indeed the cells bounding the cavities were shown to contain intact nuclei (Fig. 2f). Moreover, the isolated cavities were shown to respire at a linear rate of 2 × 10^-13 ^mol O_2 _cavity^-1 ^s^-1 ^for at least the first 15 min after being placed in fresh, oxygenated buffer, before the rate slowed over the next 45 min as oxygen was depleted in the small volume of buffer. Assuming an average leaf size of 10 cm^2 ^with 3000 cavities [4], this initial rate equates to approximately 0.5 μmol O_2 _m^-2 ^s^-1 ^at the whole leaf level - approximately 25% of whole leaf respiration, measured using a LI-6400 Portable Photosynthesis System (LiCor Environmental, Lincoln, USA).

### Isolation of intact mRNA, amplification and identification of transcripts

**Figure 3:**
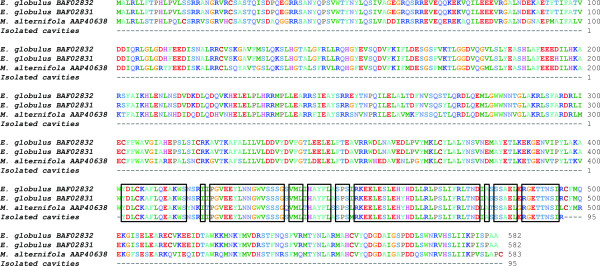
**Alignment of deduced amino acid sequences of monoterpene synthases from the Myrtaceae family**. Sequence amplified from *Eucalyptus polybractea *secretory cavity cDNA is 99% identical to two *E. globulus *monoterpene synthase sequences and 85% identical to a *Melaleuca alternifolia *sequence. Boxes delimit identical amino acids from all four sequences.

Approximately 100 ng of total RNA was obtained from 100 *E. polybractea *secretory cavities i.e. 1 ng RNA cavity^-1^. Nanodrop spectrophotometer readings gave a 260/280 nm ratio of 2.1, indicating high purity RNA was obtained. A monoterpene synthase gene and a ubiquitous actin gene (control) were successfully amplified from secretory cavity cDNA and sequenced using an AB3730xl 96-capillary sequencer (Australian Genome Research Facility Ltd, Melbourne). Specific monoterpene synthase primers amplified a 289 bp region, which Blastx (NCBI) showed to have 99% identity at the amino acid level with two monoterpene synthase sequences from *Eucalyptus globulus *and 85% identity with a putative monoterpene synthase sequence from *Melaleuca alternifolia *(GenBank accession numbers BAF02832, BAF02831 and AAP40638 respectively; Fig. 3).

### Non-essential oil constituents of secretory cavities

**Figure 4:**
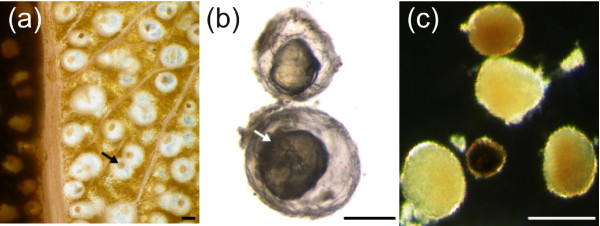
**A non-volatile component remains within the lumena of *Eucalyptus *secretory cavities after steam distillation of volatile essential oils**. (a) Steam-distilled *Eucalyptus polybractea *leaf with a portion of epidermis and attached cuticle dissected away and mesophyll cells brushed off to expose the secretory cavities. Non-volatile material remains within cavity lumena as translucent-brown material (arrow). (b) Secretory cavities of *E. globulus *(top cavity) and *E. polybractea *(bottom cavity) dissected from steam-distilled leaves and brushed free of mesophyll cells to highlight the highly abundant non-volatile material (arrow). (c) Non-volatile material extracted from cavities of steam-distilled leaves with a microprobe. All scale bars represent 100 μm.

Microscopic examination of cavities isolated with Sigma P-4716 showed that the cavity lumena contained highly abundant components that autofluoresced under UV excitation (Figs. 1f &2b). Secretory cavity lumen contents are under positive pressure [4], and puncturing the cavities resulted in the ejection of the majority of the essential oil component, but generally not the autofluorescent component (Fig. 2c), which appeared resinous after removal with a microprobe. Furthermore, lipid staining of the autofluorescent component was negative (Fig. 2c). Based on these observations, we steam-distilled fresh leaves to examine if the autofluorescent component was removed with the volatile essential oils. Interestingly, these components were found to remain in cavities from which the essential oils had been totally removed via steam distillation (Fig. 4a &4b). Moreover, these non-volatile components could be physically removed from the secretory cavities of such leaves with a microprobe (Fig. 4c).

**Figure 5:**
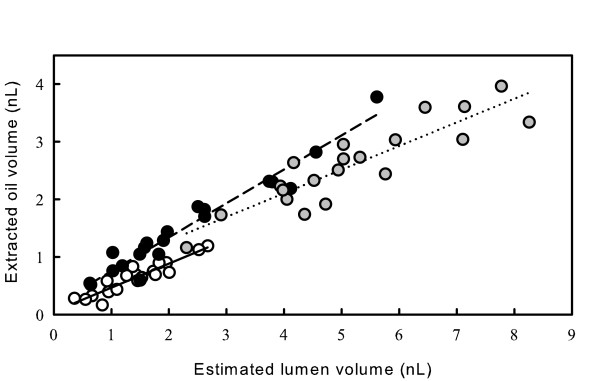
**Quantification of the abundance of volatile essential oils within the secretory cavity lumena of *Eucalyptus***. Estimated lumen volume plotted against the volume of oil extracted from each of 20 isolated cavities from fully expanded leaves of *Eucalyptus polybractea *(open circles; solid regression line), *E. globulus *(closed circles; dashed regression line) and *E. froggattii *(grey circles; dotted regression line).

We next quantified the relative abundances of the volatile essential oils and non-volatile resinous components within the lumena of secretory cavities by applying the enzymatic isolation protocol to *E. polybractea*, *E. globulus *and *E. froggattii *leaves. Cavities were isolated from each species using Sigma P-4716 and oil was extracted and quantified using gas chromatography. The total volume of volatile essential oils from each cavity was plotted against the lumen volume for that cavity, estimated by microscopic examination and imaging (Fig. 5). The linear regression for *E. polybractea *was significant (ANOVA F = 107, *P *< 0.0001) with r^2 ^= 0.86, a significant slope (± 1 s.e.) of 0.42 ± 0.04 (t = 0.8, *P *< 0.0001), but a non-significant intercept of 0.05 ± 0.06. Similarly, the linear regression for *E. globulus *was significant (ANOVA F = 324, *P *< 0.0001) with r^2 ^= 0.95, a significant slope (± 1 s.e.) of 0.59 ± 0.03 (t = 18, *P *< 0.0001), and a non-significant intercept of 0.16 ± 0.09. The linear regression for *E. froggattii *was also significant (F = 72, *P *< 0.0001) with r^2 ^= 0.80, a significant slope (± 1 s.e.) of 0.41 ± 0.05 (t = 8.5, *P *< 0.0001), and a non-significant intercept of 0.46 ± 0.26. The significant slope values for each species are relatively similar and indicate that the essential oil contents of the cavities were only 42%, 59% and 41% of the cavity lumen volume for *E. polybractea*, *E. globulus *and *E. froggattii*, respectively.

To test if the regression slopes were artificially low due to a loss of essential oils from the cavities during the isolation process, ten blocks of leaf tissue each encompassing a single cavity were hand-dissected from fully expanded leaves, imaged using a dissecting microscope with transmitted lighting and the oil extracted as for the isolated cavities. Only outer cavity diameters could be accurately estimated in the hand dissected tissue blocks therefore total cavity volume (lumen and cavity cells) was calculated, rather than lumen volume. The mean (± s.e.) extracted oil volume per unit total cavity volume for enzymatically isolated cavities was 0.22 ± 0.01 nL nL^-1 ^and for those hand-dissected in tissue blocks was 0.23 ± 0.02 nL nL^-1^. A one-way ANOVA detected no statistical difference between the means (F= 0.7, *P *= 0.41), thus it appears that no essential oils are lost during the isolation process.

Gas chromatographic analyses of the volatile oils extracted from the *Eucalyptus *secretory cavities showed the dominant constituent in *E. polybractea *and *E. globulus *cavity lumena was the oxygenated monoterpene 1,8-cineole (>50% of total essential oils), whereas *E. froggattii *cavities contained on average only 5% of this key monoterpene (Table 2). Moreover, the essential oils extracted from the secretory cavities of *E. polybractea *and *E. globulus *contained over 75% monoterpenes, but *E. froggattii *contained only 30% monoterpenes, with a much greater proportion of sesquiterpenes (Table 2). Nevertheless, as noted in Figure 5, we estimated similar relative abundances of essential oils as a proportion of the volume of secretory cavity lumena for each species.

**Table 2:** Essential oil composition (expressed as percentage of total extracted oil) for isolated secretory cavities of *Eucalyptus*^‡^.

	*E. polybractea*		*E. globulus*		*E. froggattii*	
	
Essential oil constituent (%, v/v)	mean	**s.e**.	mean	**s.e**.	mean	**s.e**.
*Hydrocarbon monoterpenes*						
α-pinene	1.2	0.1	18.7	0.4	4.5	0.2
camphene	n.d.		n.d.		1.3	0.4
β-pinene	3.0	0.3	0.2	0.1	0.5	0.1
myrcene	trace		2.2	0.1	2.8	0.1
sabinene	trace		n.d.		1.3	0.1
*p*-cymene	1.6	0.2	0.5	0.1	0.4	0.0
limonene	2.3	0.1	6.3	0.2	6.6	0.2
β-phellandrene	Trace		trace		6.7	0.2
*Oxygenated monoterpenes*						
1,8-cineole	58.7	2.8	51.6	0.8	5.0	0.3
cryptone	4.2	0.6	n.d.		n.d.	
α-terpineol	2.0	0.3	2.0	0.2	1.4	0.0
α-terpenyl acetate	1.6	0.3	trace		0.4	0.0
***Total monoterpenes***	**74.6**		**81.5**		**30.9**	
*Hydrocarbon sesquiterpenes*						
Allo-aromadendrene	trace		1.9	0.2	0.5	0.0
C_15_H_24_	trace		trace		1.4	0.0
aromadendrene	5.8	0.6	1.3	0.1	0.9	0.1
selinene	trace		2.6	0.6	0.8	0.1
C_15_H_24_	trace		trace		1.0	0.2
cadinene	2.9	0.6	n.d.		2.7	0.2
*Oxygenated sesquiterpenes*						
elemol	trace		n.d.		10.2	0.3
globulol	6.5	0.9	2.1	0.3	trace	
spathulenol	1.9	0.5	0.8	0.2	trace	
α-eudesmol	trace		trace		8.0	0.3
β-eudesmol	trace		n.d.		27.4	0.4
C_15_H_26_O	trace		n.d.		7.6	0.3
***Total sesquiterpenes***	**17.1**		**8.7**		**60.5**	
*Uncharacterised*	8.3		9.8		8.6	

^‡^Data are presented as the mean and 1 standard error (s.e.) of 20 isolated cavities for each species. Only essential oil constituents with a mean > 1.0% for any species are presented. Trace denotes mean < 0.2%, and n.d. denotes not detected.

## Discussion

The protocol described herein is able to successfully isolate intact, live secretory cavities from three *Eucalyptus *species. A method to isolate analogous alkaloid secretory cavities from leaves of marigold (Asteraceae) has previously been reported, but structural integrity was compromised during isolation resulting in a loss of between 83 and 96% of lumen contents [29]. Our protocol has the potential to be applied to help elucidate the pathways for essential oil biosynthesis as well as that of other natural products recently found in *Eucalyptus *secretory cavities [26]. We have shown that the isolated secretory cavities can be used as a source of monoterpene synthase mRNA and future work will be extended to appraise the secretory cavity transcriptome. The site of biosynthesis of essential oils in sub-dermal secretory cavities has long been presumed to be the cells bounding the cavity lumena [30], rather than elsewhere in the leaf, and the occurrence of transcript for a monoterpene synthase in the isolated cavities supports this theory. The isolated cavities are also amenable to various microscopy techniques. A number of conflicting theories have been proposed to explain the development of sub-dermal secretory cavities [8,31,32], and the application of such microscopy techniques to isolated cavities may help resolve this issue.

The use of the protocol to estimate high abundances of non-volatile resinous components within the secretory cavities of *Eucalyptus *has demonstrated the potential importance of these poorly understood constituents. In a number of studies of isolated glandular trichomes, essential oils have been found to co-occur with less volatile compounds. For example, the trichomes of species in the Lamiaceae can contain monoterpenes and non-biosynthetically related compounds such as diterpenoids in white horehound [33], phenylpropanoids in sweet basil [12], and flavone aglycones in oregano [34] and mint [35]. Nevertheless, little is known about the presence and identity of non-volatiles in sub-dermal cavities, with the exception of conifer resin ducts, which are known to contain both monoterpenes and diterpene resin acids [36]. In eucalypts, for example, it has long been assumed that the secretory cavities contain volatile essential oils alone. However, the results presented here show that up to 60% of glandular volume is allocated to a complex mixture of non-volatile components (Fig. 5). Given that *Eucalyptus globulus *is the world's major source of eucalyptus oil and *E. polybractea *is Australia's key commercial source, the high estimates of 41 and 58% non-volatile components in the cavity lumena, respectively, suggest that future research on the biosynthesis of the resinous component may have implications for increasing commercial essential oil yields.

Recently, we have shown that a large proportion of the non-volatile fraction in *Eucalyptus *secretory cavity lumena is composed of the monoterpenoid glucose esters cuniloside B and froggattiside A [26]. Notably, these compounds are not autofluorescent and the fluorescence observed in the *Eucalyptus *secretory cavity lumena described here (Figs. 1 &2) may arise from various phenolic glycosides (e.g. cypellocarpins: cypellocarpin C has recently been reported to co-occur with cuniloside B and froggattiside A in a range of *Eucalyptus *species [37]). This contention will form the basis of future studies. The biosynthesis of such non-volatile compounds and their role within the secretory cavities is not known, but the application of the isolation protocol presented here may help address these questions.

## Conclusions

Surprisingly little is known about the physiology of plant secretory structures, and in particular, those of an embedded nature. This paucity of knowledge has been ascribed to the low abundance of the cells that compose secretory structures, and the difficulty of physically isolating these cells for study [10]. The protocol described herein for isolating large numbers of intact sub-dermal secretory cavities, free from other leaf tissues, may help overcome these limiting factors for *Eucalyptus*. The protocol is likely to be adaptable to a broad range of *Eucalyptus *species with sub-dermal, foliar secretory cavities and should find application in biosynthetic studies of the numerous commercially important natural products found in eucalypt leaves.

## Methods

### Plant material

Fully expanded leaves of each species were sampled at particular life stages to maximize the abundance and size of sub-dermal secretory cavities. Secretory cavities occur in very low abundance in seedling leaves of *Eucalyptus polybractea *and *E. froggattii*, but are highly abundant in seedling leaves of *E. globulus*. Therefore *E. polybractea *leaves were sampled from plantation-grown adult trees (see [22] for plantation details) and from glasshouse-grown ramets micropropagated from the plantation trees (see [38] for micropropagation protocol). Adult *Eucalyptus froggattii *trees were sampled from a natural population (Greater Bendigo National Park, Victoria, Australia 36°30.04' S, 144°22.24' E), whereas seedlings of glasshouse-grown *E. globulus *were sampled (see [39] for seed germination and glasshouse conditions).

### Isolated cavity respiration

Sixty isolated secretory cavities were transferred to 'Standard' buffer (125 μl) and oxygen depletion in the buffer measured and logged every 2 min for 60 min using a MI-730 micro-oxygen electrode (Microelectrodes, inc. Bedford, NH) connected to an Orion 5-star meter (Thermo Fisher Scientific, Waltham, MA). A solution of 'Standard' buffer (125 μl) without secretory cavities was used as the control and control measurements were logged for 1 h before and after the isolated cavity measurements.

### Total chlorophyll determinations

Chlorophyll concentrations were determined on a per cavity basis for three leaf sections, each encompassing 50 cavities (~16 mm^2^) and also on triplicate batches of 50 isolated cavities. The leaf sections were excised from leaf strips post enzymatic digestion with Sigma P-4716 pectinase. In each case, tissue was ground in a microcentrifuge tube with a microtube pestle and extracted with 300 μl acetone (80%, v/v). Extracts were centrifuged, 250 μl of the supernatant collected and its absorbance at 647 and 664 nm measured using a Beckman DU640 spectrophotometer (Beckman Coulter, Brea, CA). Total chlorophyll per cavity was determined using the equations of Jeffrey & Humphrey (1975) [40].

### Microscopy techniques applied to isolated cavities

For confocal microscopy, isolated cavities were dehydrated in a graded series of ethanol and stained with Acid Fuchsin (0.5% v/v; Sigma) for 5 min. The cavities were then mounted in ethanol (100%) and examined by confocal fluorescence microscopy with a Leica DMIRB laser scanning microscope and a Leica TCS SP2 imaging system (Fig. 2d). For scanning electron microscopy, isolated cavities were prepared by fixation in glutaraldehyde (2.5% v/v) in standard buffer, post fixed in OsO_4 _(0.5% w/v), and dehydrated in a graded ethanol series. Cavities in 100% ethanol were then dried using a Baltec CPD 030 critical point dryer, gold-coated using an Edwards S150B sputter coater and observed with a Philips XL30 FEG Field Emission Scanning Electron Microscope (Fig. 2e). For fluorescence microscopy, cavities were mounted in standard buffer and viewed under an Olympus BH-2 microscope with UV excitation. Autofluorescence was viewed under UV or GFP2 filters (Fig. 1f &2b). Staining with Hoechst 33342 (4 μg ml^-1^; Sigma) in 'Standard' buffer for 20 min was used to visualize intact nuclei under a UV filter (Fig. 2f). Staining with the specific neutral lipid dye Nile red (10 μg ml^-1 ^in acetone; Sigma) for 10 min was used to visualize lipids under a UV filter after isolated cavities were punctured with a 1 μm microprobe (Fig. 2c).

### Lumen volume estimation and essential oil quantification

Isolated cavities were imaged on a micrometer slide under a dissecting microscope with transmitted lighting to enable visualization of the lumen through the translucent cavity epithelial cells. The lumen volume for each cavity was estimated assuming an elipsoid shape and averaging two measurements for each of the equatorial radii and the polar radius of the lumen made using ImageJ software (Version 1.42q, National Institutes of Health, USA). Each cavity was then transferred by pipette to a microtube containing two tungsten balls and 40 μl of hexane containing 100 μg ml^-1 ^tridecane as an internal standard, and ground using a Retsch MM300 mixer mill (Qiagen, Germantown, USA) for 20 s. The hexane extract was collected and analysed using a Perkin Elmer Autosystem XC GC-FID (Perkin Elmer, Melbourne, Australia) fitted with a Zebron-5 column (30 m × 0.25 mm i.d. × 0.25 μm film; Phenomenex, Torrance, USA) and with He as the carrier gas at a flow rate of 1 ml min^-1^. The column temperature was held at 70°C for 4 min following injection of a 2.5 μL aliquot, then ramped at 10°C min^-1 ^to 250°C and held for a further 4 min. Oil constituents were identified by retention time comparison with known standards (1,8-cineole, *p-*cymene, limonene, aromadendrene, terpinen-4-ol, myrcene, β-pinene, α-pinene; Sigma) or by GC-MS using a 7890A GC coupled to a 5975C mass spectrometer (Agilent Technologies, Santa Clara, USA) operated with the same column and conditions as for the GC-FID. Oil constituents were quantified by comparison with the known standards or by using the average response ratio of all compounds.

### RNA purification and cDNA synthesis

Leaf strips were incubated for 12 h with pectinase in glycerol P-4716 in a solution comprised of 50% standard buffer containing 5 mM DTT and 50% RNAlater (Ambion, Austin, TX). One hundred isolated *E. polybractea *cavities were transferred via pipette to a microfuge tube containing 100 μL of a 50:50 v/v solution of sorbital buffer and RNAlater and frozen in liquid N_2 _before being ground with a plastic microfuge pestle. Total RNA was extracted using an RNeasy Plant Mini Kit (Qiagen, Valencia, CA). RLC buffer (500 μL) with β-mercaptoethanol (5 μL) was added to the ground secretory cavity tissue and the sample was then vortexed and transferred to a QIAshredder tube. Total RNA quantity and purity (260/280 nm) was measured in a 1 μL aliquot using a NanoDrop ND-1000 spectrophotometer (Thermo Fisher Scientific). Genomic DNA was removed using DNaseI (Invitrogen, Carlsbad, CA) and cDNA was synthesised from 30 ng of RNA using SuperScript III First-Strand Synthesis SuperMix (Invitrogen) with oligo(dT)20 primers according to manufacturer's instructions.

### Primer design and PCR amplification of a putative monoterpene synthase gene

Degenerate primers 5'-TTGGAAGAGCTRSARCTATTCAC and 5'-GTTCCATCTTYTTCCATGYTKKGTC were designed based on an alignment of the published sequences of monoterpene synthase genes from *Eucalyptus globulus*, sage, rosemary and *Arabidopsis thaliana *(GenBank accession numbers AB266390 and AB266391, DQ785793 and IIAF051899, DQ839411, and AY691947, respectively). PCR conditions were as follows: 60 s at 94°C; 40 cycles of 30 s at 94°C, 60 s at 61°C, 60 s at 72°C; 300 s at 72°C. Primers specific to *E. polybractea *5'-GGTATGACTTGTGCAAAGCCT and 5'-CACCGTATTGAATTCGTGGTCT were designed using sequences obtained from PCR with degenerate primers. Actin primers 5'-ACGGCCTGGATGGCGACGTACATG and 5'-GCAGAAGGACGCCTACGTTGGTGAC for the sorghum *ac1 *actin gene (GenBank accession no. X79378) were used as a control [41]. One-twentieth of the volume of the synthesised secretory cavity cDNA was used as a template in PCR reactions using *Ex Taq *DNA polymerase (Takara, Madison, USA) with specific monoterpene synthase and actin primers. PCR conditions were as follows: 60 s at 94°C; 40 cycles of 30 s at 94°C, 30 s at 61°C, 60 s at 72°C; 300 s at 72°C.

### Statistical analyses

One-way ANOVA and regression analyses were performed using SPSS version 18.0 (SPSS Inc., Chicago, USA).

## Competing interests

The authors declare that they have no competing interests.

## Authors' contributions

JG conceived the study, developed the protocol and drafted the manuscript. AH performed the microscopy and helped to draft the manuscript. MM & EN carried out the molecular studies and helped to draft the manuscript. DK helped develop the protocol and performed the gas chromatography analyses. IW helped to design the study and draft the manuscript. All authors read and approved the final manuscript.
